# Supplementary material for: Heat adaptation of phage T7 under an extended genetic code
Source: Virus Evol. 2021 Dec 1;7(2):veab100. doi: 10.1093/ve/veab100 (PMC8923235; doi:10.1093/ve/veab100)
Supplement: veab100_Supp [file veab100_supp.zip › Sequencing_Analysis.zip!/Sequencing_Analysis/S1/evidence/JC_62.html]

BRESEQ :: Evidence


| Predicted mutation | | | | | | |
| --- | --- | --- | --- | --- | --- | --- |
| evidence | seq id | position | mutation | annotation | gene | description |
| JC | NC\_001604.1 | 27,769 | (ATATCTGGCGCT)_1→2_ | intergenic (–/–) | – / – | –/– |

| New junction evidence | | | | | | | | | | | |
| --- | --- | --- | --- | --- | --- | --- | --- | --- | --- | --- | --- |
|  | | seq id | position | reads (cov) | reads (cov) | score | skew | freq | annotation | gene | product |
| \* | ? | NC\_001604.1 | = 27753 | 15 (0.000) | 3703 (0.920) +CGCTATATCTGG | 393/544 | 0.0 | 99.6% | intergenic (–/–) | *–/–* | –/– |
|  | ? | NC\_001604.1 | 27754 = | 18 (0.000) |  |  |  |  | intergenic (–/–) | *–/–* | –/– |

Only 100 of 4277 total aligned reads displayed.  

`CTAGCTATCGGTGGTAACTGCGGGGACCAGTGCTGGTTCGTTACGAGCGACCAAGTGTGGCGACTTAGTGGAAAGGCTAAGCGAAAGTTCCGTAAGTTAATCATGGAGTATCGCGATAAGATGCTTGAGAAGTATGATACTCTTTGGAATTACGTATGGGTAGGCAATACGTCCCACATTCGTTTCCTCAAGACTATCGGTGCGGTATTCCATGAAGAGTACACACGAGATGGTCAATTTCAGTTATTTACAATCACGAAAGGAGGATAACCATATGTGTTGGGCAGCCGCAATACCTAT‑‑‑‑‑‑‑‑‑‑‑‑‑‑‑‑‑‑‑‑‑‑‑‑‑‑‑‑‑‑‑‑‑‑‑‑‑‑‑‑‑‑‑‑‑‑‑‑‑‑‑‑‑‑‑‑‑‑‑‑‑‑‑‑‑‑‑‑‑‑‑‑‑‑‑‑‑‑‑‑‑‑‑‑‑‑‑‑‑‑‑‑‑‑‑‑‑‑‑‑‑‑‑‑‑‑‑‑‑‑‑‑‑‑‑‑‑‑‑‑‑‑‑‑‑‑‑‑‑‑‑‑‑‑‑‑‑‑‑‑‑‑‑‑‑‑‑‑‑‑‑‑‑‑‑‑‑‑‑‑‑‑‑‑‑‑‑‑‑‑‑‑‑‑‑‑‑‑‑‑‑‑‑‑‑‑‑‑‑‑‑‑‑‑‑‑‑‑‑‑‑‑‑‑‑‑‑‑‑‑‑‑‑‑‑‑‑‑‑‑‑‑‑‑‑‑‑‑‑‑‑‑‑‑‑‑‑‑‑‑‑‑‑‑‑‑‑‑‑‑‑‑‑‑‑‑‑‑‑‑‑‑‑‑‑‑‑‑‑‑‑‑‑‑‑‑‑‑‑‑‑‑‑‑‑‑‑‑‑‑‑‑‑‑‑‑‑‑‑‑‑‑‑‑‑‑‑‑‑‑‑‑  >  NC_001604.1/27454‑27753`  
`‑‑‑‑‑‑‑‑‑‑‑‑‑‑‑‑‑‑‑‑‑‑‑‑‑‑‑‑‑‑‑‑‑‑‑‑‑‑‑‑‑‑‑‑‑‑‑‑‑‑‑‑‑‑‑‑‑‑‑‑‑‑‑‑‑‑‑‑‑‑‑‑‑‑‑‑‑‑‑‑‑‑‑‑‑‑‑‑‑‑‑‑‑‑‑‑‑‑‑‑‑‑‑‑‑‑‑‑‑‑‑‑‑‑‑‑‑‑‑‑‑‑‑‑‑‑‑‑‑‑‑‑‑‑‑‑‑‑‑‑‑‑‑‑‑‑‑‑‑‑‑‑‑‑‑‑‑‑‑‑‑‑‑‑‑‑‑‑‑‑‑‑‑‑‑‑‑‑‑‑‑‑‑‑‑‑‑‑‑‑‑‑‑‑‑‑‑‑‑‑‑‑‑‑‑‑‑‑‑‑‑‑‑‑‑‑‑‑‑‑‑‑‑‑‑‑‑‑‑‑‑‑‑‑‑‑‑‑‑‑‑‑‑‑‑‑‑‑‑‑‑‑‑‑‑‑‑‑‑‑‑‑‑‑‑‑‑‑‑‑‑‑‑‑‑‑‑‑‑‑‑‑‑‑‑‑‑‑‑‑‑‑‑‑‑‑‑‑‑‑‑‑‑‑‑‑‑‑‑‑‑‑CGCTATATCTGGCGCTCAGGCTATCAGTGGTCAGAACGCTCAGGCCAAAATGATTGCCGCTCAGACCGCTGCTGGTCGTCGTCAAGCTATGGAAATCATGAGGCAGACGAACATCCAGAATGCTGACCTATCGTTGCAAGCTCGAAGTAAACTTGAGGAAGCGTCCGCCGAGTTGACCTCACAGAACATGCAGAAGGTCCAAGCTATTGGGTCTATCCGAGCGGCTATCGGAGAGAGTATGCTTGAAGGTTCCTCAATGGACCGCATTAAGCGAGTCACAGAAGGACAGTTCATTCGGGA  >  NC_001604.1/27754‑28053`  

                                                                                                                                                                                                                                                                                                            `||||||||||||`                                                                                                                                                                                                                                                                                                              
 `ggagTATCGTTGGTACCAGCGGGAACCAGTGCCGGTTCATTACGGGCGACAAAGTGTGGCGACTTGGTGGAAAGACTAAGCGAAGGATCCGTAAGTTAATCATGGAGTATCGCGATAAGATGCTTGAGAAGTATGCTCCTCTTTGGAATTACGTATGGCTAGGCAATACGTCCCACATTCGTTTCCTCAAGACTATCGGTGCGGTATTCCATAAAGAGTACACACGAGATGGTCAATTTCAGTTATTTACAATCACGAAAGGAGGATAACCATATGTGTTGGGCAGCCGCAATACCTATCG                                                                                                                                                                                                                                                                                                                        <  1:403993/297‑1`  
  `AGCTATCGGTGGTAACTGCGGGGACCAGTGCTGGTTCGTTACGAGCGACCAAGTGTGGCGACTTAGTGGAAAGGCTAAGCGAAAGTTCCGTAAGTTAATCATGGAGTATCGCGATAAGATGCTTGAGAAGTATGATACTCTTGGGAATTACGTATGGGTAGGCAATACGTCCCACATTCGTTTCCTCAAGACTATCGGTGCGGTATTCCATGAAGAGTACACACGAGATGGTCAATTTCAGTTATTTACAATCACGAAAGGAGGATAACCATATGTGTTGGGCAGCCGCAATACCTATCGC                                                                                                                                                                                                                                                                                                                       <  1:81130/301‑1`  
            `GGTAACTGCGGGGACCAGTGCTGGTTCGTTACGAGCGACCAAGTGTGGCGACTTAGTGGAAAGGCTAAGCGAAAGTTCCGTAAGTTAATCATGGAGTATCGCGATAAGATGCTTGAGAAGTATGATACTCTTTGGAATTACGTATGGGTAGGCAATACGTCCCACATTCGTTTCCTCAAGACTATCGGTGCGGGATTCCATGCAGAGTACACACGAGATGGTCAATTTCAGTTATTTACAATCTCGAAATGAGGATAACCATATGTGTTGGGCAGCCGCAATCCCTATCGATATATATGGC                                                                                                                                                                                                                                                                                                             >  1:658250/1‑301`  
                        `GACCAGTGCTTGTTAGGTACTAGCGACCAAGTGTTGCGTCTTAGTGGAAAGGCTAAGCGAAAGTTCCGTAAGTTAATCATGGAGTATCGCGATAAGATGCTTGAGAAGTATGATACTCTTTGGAATTACGTATGGGTATGCAATACGTCCTACATTCTTTTCCTCAAGACTATCGGTTCGGTATTCCATGAAGAGTACACACGAGATGGTCAATTTCAGTTATTTACAATCACGAAAGGAGGATAACCATATGTGTTGGGCAGCCGCAATACCTATCGCTATATCTGGCGCTATATCTGGC                                                                                                                                                                                                                                                                                                 <  1:617268/301‑1`  
                        `GACCAGTGCTGGTTCGTTACGAGCGACCAAGTTTTGCGACTTCTTGGAACGGCTAAGCGAAAGTTCCGTACGTTAATCATGGAGTATCGCGATAAGATGCTTGAGAAGTATGATACTCTTTGCAATTACGTATGGGTAGGCAATACGTCCCACATTCGTTTCCTCCAGACTATCGGTGCGGTATTCCATGAAGAGTACACACGATCTGTTCCATTTCAGTTATTTCCACTCACGAAATGAGGATAACCATATGTGTTGGGCAGCCGCAATACCTATCGCTATCTCTGGCGCTATATCTTGC                                                                                                                                                                                                                                                                                                 >  1:471151/1‑301`  
                        `GACCAGTGCTGGTTCGTTACGAGCGACCAAGTGTGGCGACTTAGTGGAAAGGCTAAGCGAAAGTTCCGTAAGTTAATCATGGAGTATCGCGATAAGATGCTTGAGAAGTATGATACTCTTTGGAATTACGTATGGGTAGGCAATACGTCCCACATTCGTTTCCTCAAGACTATCGGTGCGGTATTCCATGAAGAGTACACACGAGATGGTCAATTTCAGTTATTTACAATCACGAAAGGAGGATAACCATATGTGTTGGGCAGCCGCAATACCTATCGCTATATCTGGCGCTATATCTGGC                                                                                                                                                                                                                                                                                                 <  1:334916/301‑1`  
                          `CCAGTGCTGGTTCGTTACGAGCGACCAAGTGTGGCGACTTAGTGGAAAGGCTAAGCGAAAGTTCCGTAAGTTAATCATGGAGTATCGCGATAAGATGCTTGAGAAGTATGATACTCTTTGGAATTACGTATGGGTAGGCAATACGTCCCACATTCGTTTCCTCAAGACTATCGGTGCGGTATTCCATGAAGAGTACACACGAGATGGTCAATTTCAGTTATTTACAATCACGAAAGGAGGATAACCATATGTGTTGGGCAGCCGCAATACCTATCGCTATATCTGGCGCTATATCTGGCGC                                                                                                                                                                                                                                                                                               >  1:483412/1‑301`  
                             `GTGCTGGTTCGTTACGAGCGACCAAGTGTGGCGACTTAGTGGAAAGGCTAAGCGAAAGTTCCGTAAGCTAATCATGGAGTATCGCGATAAGATGCTTGAGAAGTATGATACTCTTTGGAACTACGTATGGGTAGGCAATACGTCCTACATTCGTTTCCTCAAGACTATCGGTGCGGTAGTTCATGAAGAGTACAAACGAGATGGTCACTTTCAGTTATTTACAATTACTAAAGGAGGATAACCTTATGTGTTCGGCAGCCGCAATACATATCGCTCTATCTGCCGCTCTATCTGGCGCTCA                                                                                                                                                                                                                                                                                            >  1:220437/1‑301`  
                                              `gaGACCAATTGGGGAGACATAGTGGAAAGTCTAAGCGAAAGTTACGTAAGTTAATCAAGGAGTATCGAGATAAGGTGCATGAGAAGTATGATACGCGTTGGAATTACGTATGGGTAGGCAATACGTCCCACATTCGTTTCCACACGACTATCAGTGCCGTATGCCATAAAGAGTACACACGAGATGGTCAATTTCAGTTATTTACAATCACGAAAGGAGGATAACCATATGTGTTGGGCAGCCGCAATACCTATCGCTATATCTGGCGCTATATCTGGCGCTCAGGCTATCAGTGGTCAGA                                                                                                                                                                                                                                                                           <  1:611139/299‑1`  
                                                           `GCGACTTAGTGGAAAGGCTAAGCGAAAGTTCCGTAAGTTAATCATGGAGTATCGCGATAAGATGCTTGAGAAGTATGATACTCTTTGGAATTACGTATGGGTAGGCAATACGTCCCACATTCGTTTCCTCAAGACTATCGGTGCGGTATTCCATGAAGAGTACACACGAGATGGTCAATTTCAGTTATTTACAATCACGAAAGGAGGATAACCATATGTGTTGGGCAGCCGCAATACCTATCGCTATATCTGTCGCGATATCTGGCGCTCAGGCTATCCGTGGTCAGAACGCTCAGGCCAA                                                                                                                                                                                                                                                              >  1:438561/1‑301`  
                                                                 `aagggtAAAGGCAAATCGAAAGTTCCGTAATTTAATCAAGGAGTATCACAATAATATGCTTTAAAAGTATGATACTCATTAGCATTACGTATGGGTAGGCAATACGTCCCACATTCGTTTCCTCAAGACTATCGGTGCGGTATTCCATGAAGAGTACACACGAGATGGTCAATTTCAGTTATTTACAATCACGAAAGGAGGATAACCATATGTGTTGGGCAGCCGCAATACCTATCGCTATATCTGGCGCTATATCTGGCGCTCAGGCTATCAGTGGTCAGAACGCTCAGGCCAAAATGAT                                                                                                                                                                                                                                                        <  1:279615/295‑1`  
                                                                                        `ttCGTAAGTTAATCATGGAGTATCGCGATAAGATGCTTGAGAAGTATGATACTCTTTGGAATTACGTATGGGTAGGCAATACGTCCCACATTCGTTTCCTCAAGACTATCGGTGCGGTATTCCATGAAGAGTACACACGAGATGGTCAATTTCAGTTATTTACAATCACGAAAGGAGGATAACCATATGTGTTGGGCAGCCGCAATACCTATCGCTATATCTGGCGCTATATCTGGCGCTCAGGCTATCAGTGGTCAGAACGCTCAGGCCAAAATGATTGCCGCTCAGACCGCTGCTGGTC                                                                                                                                                                                                                                 <  1:69801/299‑1`  
                                                                                          `CGTAAGTTAATCATGGAGTATCGCGATAAGATGCTTGAGAAGTATGATACTCTTTGGAATTACGTATGGGTAGGCAATACGTCCCACATTCGTTTCCTCAAGACTATCGGTGCGGTATTCCATGAAGAGTACACACGAGATGGTCAATTTCAGTTATTTACAATCACGAAAGGAGGATAACCATATGTGTTGGGCAGCCGCAATACCTATCGCTATATCTGGCGCTATATCTGGCGATCAGGCTATCAGTGGTCAGAACGCTCAGGCCAAAATGATTGCCGCTCAGACCGCTGCTGGTCGT                                                                                                                                                                                                                               <  1:541421/301‑1`  
                                                                                                 `TAATCATGGAGTATCGCGATAAGATGCTTGAGAAGTATGATACTCTTTGGAATTACGTATGGGTAGGCAATACGTCCCACATTCGTTTCCTCAAGACTATCGGTGCGGTATTCCATGAAGAGTACACACGAGATGGTCAATTTCAGTTATTTACAATCACGAAAGGAGGATAACCATATGTGTTGGGCAGCCGCAATACCTATCACTATATCTGGCGCTATATCTGGCGCTCAGGCTATCAGTGGTCAGAACGCTCAGGCCAAAATGATTGCCGCTCAGACCGCTGCTGGTCGTCGTCAAG                                                                                                                                                                                                                        >  1:476840/1‑301`  
                                                                                                  `AATCATGGAGTATCGCGATAAGATGCTTGAGAAGTATGATACTCTTGGGAATTACGTATGGGTAGGCAATACGTCCCACATTCGTTTCCTCAAGACTATCGGTGCGGTATTCCATGAAGAGTACACACGAGATGGTCAATTTCAGTTATTTACAATCACGAAAGGAGGATAACCATATGTGTTGGGCAGCCGCAATACCTATCGCTATATCTGGCGCTATATCTGGCGCTCAGGCTATCAGTGGTCAGAACGCTCAGGCCAAAATGATTGCCGCTCAGACCGCTGCTGGTCGTCGTCAAGC                                                                                                                                                                                                                       <  1:274923/301‑1`  
                                                                                                   `ATCATGGAGTATCTCGATAAGATGCTTGAGAAGTATGATACTCTTTGGAATTACGTATGGGTAGGCAATACGTCACACATTCGTTTCCTCAAGACTATCGGTGCGGTATTCCATGAAGAGTACACACGAGATGGTCAATTTCAGTTATTTACAATCACGAAAGGAGGATAACCATATGTGTTGGGCAGCCGCAATACCTATCGCTATATCTGGCGCTATATCTGGCGCTCAGGCTATCAGTGGTAAGAACGCTCAGGCCAAAATGATTTCCGCTCAGACCGCTGCTGGTCGTCGTCAAGCT                                                                                                                                                                                                                      >  1:345091/1‑301`  
                                                                                                   `ATCATGGAGTATCGCGATAAGATGCTTGAGAAGTATGATACTCTTTGGAATTACGTATGGGTAGGCAATACGTCCCACATTCGTTTCCTCAAGACTATAGGTGCGGTATTCCATGAAGAGTACACACGAGATGGTCAATTTCAGTTATTTACAATCACGAAAGGAGGATAACCATATGTGTTGGGCAGCCGCAATACCTATCGCTATATCTGGCGCTATATCTGGCGCTCAGGCTATCAGTGGGCAGAACGCTCAGGCCAAAATGATTGCCGCTCAGACCGCTGCTGGTCGTCGTCAAGCT                                                                                                                                                                                                                      >  1:254300/1‑301`  
                                                                                                   `ATCATGGAGTATCGCGATAAGATGCTTGAGAAGTATGATACTCTTTGGAATTACGTATGGGTAGGCAATACGTCCCACATTCGTTACCTCAAGACTATCGGTGCGGTATTCCATGAAGAGTACACACGAGATGGTCAATTTCAGTTATTTACAATCACGAAAGGAGGATAACCATATGGTTTGGGCAGCCGCAATACCTACCGCAATATCTGGCGCTATATCTGGCGCACGGCCTTTAAGTGGTAGAAACCCCAAGGCCAAATTGATGGCCGCTCAGCCCGCTCTTGGTCtccgcaaacct                                                                                                                                                                                                                      >  1:303365/1‑290`  
                                                                                                      `ATGGAGTATCGCGATAAGATGCTTGAGAAGTATGATACTCTTTGGAATTACGTATGGGTAGGCAATACGTCCCACATTCGTTTCCTCAAGACTATCGGTGC‑GTATTCCATGAAGAGTACACACGAGATGGTCAATTTCAGTTATTTACAATCACGAAAGGAGGATAACCATATGTGTTGGGCAGCCGCAATACCTATCGCTATATCTGGCGCTATATCTGGCGCTCAGGCTATCAGTGGTCAGAACGCTCAGGCCAAAATGATTGCCGCTCAGACCGCTGCTGGTCGTCGTCAAGCTATGG                                                                                                                                                                                                                  >  1:192672/1‑301`  
                                                                                                               `acaGATAAGATGCTTGAGAAGTATGATACTCTTTGGAATTACGTATGGGTAGGCAATACGTCCCACATTCGTTTCCTCAAGACTATCGGTGCGGTATTCCATGAAGAGTACACACGAGATGGTCAATTTCAGTTATTTACAATCACGAAAGGAGGATAACCATATGTGTTGGGCAGCCGCAATACCTATCGCTATATCTGGCGCTATATCTGGCGCTCAGGCTATCAGTGGTCAGAACGCTCAGGCCAAAATGATTGCCGCTCAGACCGCTGCTGGTCGTCGTCAAGCTATGGAAATCATG                                                                                                                                                                                                          <  1:426246/298‑1`  
                                                                                                                       `GATGCTTGAGAAGTATGATACTCTTTGGAATTACGTATGGGTAGGCAATACGTCCCACATTCGTTTCCTCAAGACTATCGGTGCGGTATTCCATGAAGAGTACACACGAGATGGTCAATTTCAGTTATTTACAATCACGAAAGGAGGATAACCATATGTGTTGGGCAGCCGCAATACCTATCGCTATATCTGGCGCTATATCTGGCGCTCAGGCTATCAGTGGTCAGAACGCTCAGGCCAAAATGATTGCCGCTCAGACCGCTGCTGGTCGTCGTCAAGCTATGGAAATCATGAGGCAGAC                                                                                                                                                                                                  >  1:641683/1‑301`  
                                                                                                                        `agagaaagGAAGGATGATATGCTTTGGAATTACGTATGGATAGGCAATACGTCACACATGCGTTTCCTCAAGACTATCGGTGCAGTATTCCATGAAGAGTACACACGAGATGGTCAATTGCAGTTATTTACAATCACGAAAGGAGGATAACCATATGTGTTGGGCAGCCGCAATACCTATCGCTATATCTGGCGCTATATCTGGCGCTCAGGCTATCAGTGGTCAGAACGCTCAGGCCAAAATGATTGCCGCTCAGACCGCTGCTGGTCGTCGTCAAGCTATGGAAATCATGAGGCAGACG                                                                                                                                                                                                 <  1:350024/293‑1`  
                                                                                                                        `ATGCTTGAGAAGTATGATACTCTTTGGAATTACGTATGGGTAGGCAATACGTCCCACATTCGTTTCCTCAAGACTATCGGTGCGGTATTCCATGAATAGTACACCCGAGATGGTCAATTTCAGTTATTTACAATCACGTAAGGTGGATTACCATCTGTGTTGGCCAGCCGCAATACTTATCGCTATATCTGGCGCTATATCTTGCGTTCAGGCTTTCACTGGTCAGATCGCTCAGGCCAACATGATTGCCGCTCAGACCGCTGCTGTTCGTCGTCCAGCTATGGAAATCTTGAGGCTGAtg                                                                                                                                                                                                 >  1:554167/1‑299`  
                                                                                                                               `gGAAGTATGATACTCTTTGTAATTACGTATGGGTAGGCAATACGTCCCACATTCGTTTCCTCAAGACTATCGGTGCGGTATTCCATGAAGAGTACACACGAGATGGTCAATTTCAGTTATTTACAATCACGAAAGGAGGATAACCATATGTGTTGGGCAGCCGCAATACCTATCGCTATATCTGGCGCTATATCTGGCGCTCAGGCTATCAGTGGTCAGAACGCTCAGGCCAAAATGATTGCCGCTCAGACCGCTGCTTGTCGTCGTCAAGCTATGGAAATCATGAGGCAGACGAACATCC                                                                                                                                                                                          <  1:105041/300‑1`  
                                                                                                                                 `agGTATGATACTCTTTGGAATTACGTATGGGTAGGCAATACGTCCCACATTCGTTTCCTCAAGACTATCGGTGCGGTATTCCATGAAGAGTACACACGAGATGGTCAATTTCAGTTATTTACAATCACGAAAGGAGGATAACCATATGTGTTGGGCAGCCGCAATACCTATCGCTATATCTGGCGCTATATCTGGCGCTCAGGCTATCAGTGGTCAGAACGCTCAGGCCAAAATGATTGCCGCTCAGACCGCTGCTGGTCGTCGTCAAGCTATGGAAATCATGAGGCAGACGAACATCCAG                                                                                                                                                                                        <  1:197280/299‑1`  
                                                                                                                                   `GTATGATACTCTTTGGAATTACGTTTGTGTAGGCAATACGTCCCACATTCGTTTCCTCAAGACTATCGGTGCGGTATTCCATGAAGAGTACACATGAGATGGTCAATTTCAGTTATCTACAATCACGAAAGGAGGATAACCATATGTGTTGGGCAGCCGCCATACCTATCGCTATATATGGCGATATATCTGGCGCTCAGGCTATCAGTGGTCAGAACGCACAGGCCACAATGATTACAGCTCAGACCGCTGCTGGTCGTCGTCAAGATATGGAACTCATGAGGCAGACGAACATCCAGAA                                                                                                                                                                                      >  1:598985/1‑301`  
                                                                                                                                     `ATGATACTCTTTGGAATTACGTATGGGTAGGCAATACGTCCCACATTCGTTTCCTCAAGACTATCGGTGCGGTATTCCATGAAGAGTACACACGAGATGGTCAATTTCAGTTATTTACAATCACGAAAGGAGGATAACCATATGTGTTGGGCAGCCGCAATACCTATCGCTATATCTGGCGCTATATCTGGCGCTCAGGCTATCAGTGGTCAGAACGCTCAGGCCAAAATGATTGCCGCTCAGACCGCTGCTGGTCGTCGTCAAGCTATGGAAATCATGAGGCAGCCGAACATCCAGAATG                                                                                                                                                                                    >  1:159776/1‑301`  
                                                                                                                                      `TGATACTCTGTGTAATTACGTAGGGGTAGGCAATACGTCCCACATTCGTTTCCTAAAGACTATCGGTGCGGTATGCCATGAAGAGTACACACGAGATGGTCAATTTCAGTTATTTACAATCACGAAAGGAGGATAACCATATGTGTTGGGCAGCCGCAATACCTATCGCTATATCTGGCGCTATATCTGGCGCTCAGGCTATCAGTGGTCAGAACGCTCAGGCCAAAATGATTGCCGCTCAGACCGCTGCTGGTCGTCGTCAAGCTATGGAAATCATGAGGCAGACGAACATCCAGAATGC                                                                                                                                                                                   <  1:622975/301‑1`  
                                                                                                                                          `cCTCTTTAGTATTACGTATGAGTCAGCAATACCTCCCACATTCGTTTCCTCAAGCCTATCCGTGCGGTATTCCATGAAGAGTACACTCGAGATGGTCAATTTCAGATAATTACAATCACGAAAGGAGGATAACCATATGTGTTGGGCAGCCGCAATACCTATCGCTATATCTAGCGATATATCTGGCGCTCAGGCTATCAGTGGTCAGAACGCTCAGGCCAAAATGATTGCCGCTCAGACCGCTGCTAGTCGTCGTCAAGCTATGGAAATCATGAGGCAGACGAACATCCAGAATGCTGAC                                                                                                                                                                               <  1:560861/300‑1`  
                                                                                                                                           `CTCTTTGGAATTACGTATGGGTAGGCAATACGTCCCACATTCGTTTCCTCAAGACTATCGGTGCGGTATTCCATGAAGAGTACACACGAGATGGTCAATTTCAGTTATTTACAATCACGAAAGGAGGATAACCATATGTGTTGGGCAGCCGCAATACCTATCGCTATATCTGGCGCTATATCTGGCGCTCAGGCTATCAGTGGTCAGAACGCTCAGGCCAAAATGATTGCCGCTCATACCGCTGCTGGTCGTCGTCAAGCTATGGAAATCATGAGGCAGACGAACATCCAGAATGCTGACC                                                                                                                                                                              <  1:529312/301‑1`  
                                                                                                                                           `CTCTTTGGAATTACGTATGGGTAGGCAATACGTCCCACATTCGTTTCCTCAAGACTATCGGTGCGGTATTCCATGAAGAGTACACACGAGATGGTCAATTTCAGTTATTTACAATCACGAAAGGAGGATAACCATATGTGTTGGGCAGCCGCAATACCTATCGCTATATCTGGCGCTATATCTGGCGCTCAGGCTATCAGTGGTCAGAACGCTCAGGCCAAAATGATTGCCGCTCAGACCGCTGCTGGTCGTCGTCAAGCTATGGAAATCATGAGGCAGACGAACATCCAGAATGCTGACC                                                                                                                                                                              <  1:147942/301‑1`  
                                                                                                                                                     `agACGTATTGGTAGGCAATACGTCCCACATTCGTTTCCTCAAGACTATCGGTGCGGTATTCCATGAAGAGTACACACGAGATGGTCAATTTCAGTTATTTACAATCACGAAAGGAGGATAACCATATGTGTTGGGCAGCCTCACTACCTATCGCTATATCTGGCGCTATATCTGGCGCTCAGGCTATCAGTGGTCAGAACGCTCAGGCCAAAATGATTGCCGCTCAGACCGCTGCTGGTCGTCGTCAAGCTATGGAAATCATGAGGCAGACGAACATCCAGAATGCTGACCTATCGTTGCA                                                                                                                                                                    <  1:445584/299‑1`  
                                                                                                                                                     `TTACGTATGGGTAGGAAATACGTCCCACATTCGTTTCCTCAAGACTATCGGTGCGGTATTCCATGAAGAGTACACACGAGATGGTCAATTTCAGTTATTTACAATCACGAAAGGAGGATAACCATATGTGTTGGGCAGCCGCAATACCTATCGCTATATCTGGCGCTATATCTGGCGCTCAGGCTATCAGTGGTCAGAACGCTCAGGCCAAAATGATTGCCGCTCAGACCGCTGCTGGTCGTCGTCAAGCTATGGAAATCATGAGGCAGACGAACATCCAGAATGCTGACCTATCGTTGCA                                                                                                                                                                    <  1:466090/301‑1`  
                                                                                                                                                       `ACGTATGGGTAGGCAATACGTCCCACATTCGTTTCCTCAAGACTATCGGTGCGGTATTCCATGAAGAGTACACACGAGATGGTCAATTTCAGTTATTTACAATCACGAAAGGAGGATAACCATATGTGTTGGGTAGCCGCAATACCTATCGCTATATCTGGCGCTATATCTGGCGCTCAGGCTATCAGTGGTCAGAACGCTCAGGCCAAAATGATTGCCGCTCAGACCGCTGCTGGTCGTCGTCCATCTATGGCAATCATTATGCAGACGCACATCCAGAATCCTTACCTATCGTTGCAAa                                                                                                                                                                  >  1:74368/1‑300`  
                                                                                                                                                       `ACGTATGGGTAGGCAATACGTCCCACATTCGTTTCCTCAAGACTATCGGTGCGGTATTCCATGAAGAGTACACACGAGATGGTCAATTTCAGTTATTTACAATCACGAAAGGAGGATAACCATATGTGTTGGGCAGCCGCAATACCTATCGCTATATCTGGCGCTATATCTGGCGCTCAGGCTATCAGTGGTCAGAACGCTCAGGCCAAAATGATTGCCGCTCAGACCGCTGCTGGTCGTCGTCAAGCTATGGAAATCATGAGGCAGACGAACATCCAGAATGCctgtctcttatacacat                                                                                                                                                                  >  1:154460/1‑284`  
                                                                                                                                                       `ACGTATGGGTAGGCAATACGTCCCACATTCGTTTCCTCAAGACTATCGGTGCGGTATTCCATGAAGAGTACACACGAGATGGTCAATTTCAGTTATTTACAATCACGAAAGGAGGATAACCATATGTGTTGGGCAGCCGCAATACCTATCGCTATATCTGGCGCTATATCTGGCGCTCAGGCTATCAGTGGTCAGAACGCTCAGGCCAAAATGATTGCCGCTCAGACCGCTGCTGGTCGTCGTCAAGCTATGGAAATCATGAGGCAGACGAACATCCAGAATGCTGACCTATCGTTGCAAG                                                                                                                                                                  >  1:604088/1‑301`  
                                                                                                                                                       `ACGTATGGGTAGGCAATACGTCCCACATTCGTTTCCTCAAGACTATCGGTGCGGTATTCCATGAAGAGTACACACGAGATGGTCAATTTCAGTTATTTACAATCACGAAAGGAGGATAACCATATGTGTTGGGCAGCCGCAATACCTATCGCTATATCTGGCGCTATATCTGGCGCTCAGGCTATCAGTGGTCAGAACGCTCAGGCCAAAATGATTGCCGCTCAGACCGCTGCTGGTCGTCGTCAAGCTATGGAAATCATGAGGCAGACGAACATCCAGAATGCTGACCTATCGTTGCAAG                                                                                                                                                                  >  1:586414/1‑301`  
                                                                                                                                                                `aAGGCAATACGTCCCACATTCGTTTCCTCAAGACTATCTGTGCGGTATTCCATGAAGAGTACACACGAGATGGTCAATGTCAGTTATTTACAATCACGAAAGGAGGATAACCATATGTGTTGGGCAGCCGCAATACCTATCTCTATATCTGGCGCTATATCTGGCGCTCAGGCTATCAGTGGTCAGAACGCTCAGGCCAAAATGATTGCCGCTCAGACCGCTGCTGGTCGTCGTCAAGCTATGGAAATCATGAGGCAGACGAACATCCAGAATGCTGACCTATCGTTGCAAGCTCGAAGTA                                                                                                                                                         <  1:269029/300‑1`  
                                                                                                                                                                 `AGGCAATACGTCCCACATTCGTTTCCTCAAGACTATCGGTGCGGTATTCCATGAAGAGTACACACGAGATGGTCAATTTCAGTTATTTACAATCACGAAAGGAGGATAACCATATGTGTTGGGCAGCCGCAATACCTATCGCTATATCTGGCGCTATATCTGGCGCTCAGGCTATCAGTGGTCAGAACGCTCAGGCCAAAATGATTGCCGCTCAGACCGCTGCTGGTCGTCGTCAAGCTATGGAAATCATGAGGCAGACGAACATCCAGAATGCTGCCCTATCGTTGCAAGCTCGAAGctg                                                                                                                                                        >  1:263069/1‑298`  
                                                                                                                                                                           `TCCCACATTCGTTTCCTCAAGACTATCGGTGCGGTATTCCATGAAGAGTACACACGAGATGGTCAATTTCAGTTATTTACAATCACGAAAGGAGGATAACCATATGTGTTGGGCAGCCGCAATACCTATCGCTATATCTGGCGCTATATCTGGCGCTCAGGCTATCAGTGGTCAGAACGCTCAGGCCAAAATGATTGCCGCTCAGACCGCTGCTGGTCGTCGTCAAGCTATGGAAATCATGAGGCAGACGAACATCCAGAATGCTGACCTATCGTTGCAAGCTCGAAGTAAACTctgtctc                                                                                                                                              >  1:592288/1‑294`  
                                                                                                                                                                           `TCCCACATTCGTTTCCTCAAGACTATCGGTGCGGTATTCCATGAAGAGTACACACGAGATGGTCAATTTCAGTTATTTACAATCACGAAAGGAGGATAACCATATGTGTTGGGCAGCCGCAATACCTATCGCTATATCTGGCGCTATATCTGGCGCTCAGGCTATCAGTGGTCAGAACGCTCAGGCCAAAATGATTGCCGCTCAGACCGCTGCTGGTCGTCGTCAAGCTATGGAAATCATGAGGCAGACGAACATCCAGAATGCTGACCTATCGTTGCAAGCCCGAAGTAAACTTGATGAA                                                                                                                                              >  1:653384/1‑301`  
                                                                                                                                                                                `ggctcggagatgtgtataagagacaGCGGTATTCCATGAAGAGTACACACGAGATGGTCAATTTCAGTTATTTAAAATCACGAAAGGAGGATAACCATATGTGTTGGGCAGCCGCAATACCTATCGCTATATCTGGCGCTATATCTGGCGCTCAGGCTATCAGTGGTCAGAACGCTCAGGCCAAAATGATTGCCGCTCAGACCGCTGCTGGTCGTCGTCAAGCTATGGAAATCATGAGGCAGACGAACATCCAGAATGCTGACCTATCGTTGCAAGCTCGAAGTAAACTTGAGGAAGCGTC                                                                                                                                         <  1:128495/276‑1`  
                                                                                                                                                                                         `CCTCAAGACTATCGGTGCGGTATTCCATGAAGAGTACACACGAGATGGTCAATTTCAGTTATTTACAATCACGAAAGGAGGATAACCATATGTGTTGGGCAGCCGCAATACCTATCGCTATATCTGGCGCTATATCTGGCGCTCAGGCTATCAGTGGTCAGAACGCTCAGGCCAAAATGATTGCCGCTCAGACCGCTGCTGGTCGTCGTCAAGCTATGGAAATCATGAGGCAGACGAACATCCAGAATGCTGACCTATCGTTGCAAGCTCGAAGTAAACTctgtctcttatacacatctcc                                                                                                                                >  1:340227/1‑280`  
                                                                                                                                                                                         `CCTCAAGACTATCGGTGCGGTATTCCATGAAGAGTACACACGAGATGGTCAATTTCAGTTATTTACAATCACGAAAGGAGGATAACCATATGTGTTGGGCAGCCGCAATACCTATCGCTATATCTGGCGCTATATCTGGCGCTCAGGCTATCAGTGGTCAGAACGCTCAGGCCAAAATGATTGCCGCTCAGACCGCTGCTGGTCGTCGTCAAGCTATGGAAATCATGAGGCAGACGAACATCCAGAATGCTGACCTATCGTTGCAAGCCCGAAGGAAACTTGAGGAAGCGTCCGCCCAGTT                                                                                                                                >  1:142455/1‑301`  
                                                                                                                                                                                          `CTCAAGACTATCGGTGCGGTATTCCATGAAGAGTACACACGAGATGGTCAATTTCAGTTATTTACAATCACGAAAGGAGGATAACCATATGTGTTGGGCAGCCGCAATACCTATCGCTATATCTGGCGCTATATCTGTCGCTCAGGCTATCAGTGGTCAGAACGCTCAGGCCAAAATGATTGCCGCTCAGACCGCTGCTGGTCGTCGTCAAGCTATGGAAATCATGAGGCAGACGAACATCCAGAATGCTGACCTATCGTTGCAAGCTCGAAGTAAACTTGAGGAAGCGTCCGCCGAGTTG                                                                                                                               >  1:113070/1‑301`  
                                                                                                                                                                                            `CAAGACTATCGGTGCGGTATTGCATGAAGAGTACACACGAGATGGACAATTTCAGTTATGTACAATCACGAAAGGAGGATAACCATATGTGTTGGGCAGCCGCAATAACTATCGCTATATCTGGCGCTATATCTGGCGCTCGGGCTATCAGTGGTCAGAACGCTCAGGCTAAAATGATTGCCGCTCAGACAGCTGCTGGTCGTCGTCAAGCTATGGAAATCATGAGGCATACGAAAATCCAGAATGCTGACTCATCGTTGCAAGCTCGAAGTAAACGTGAGGAAGCGTCCGCCGAGTTGAC                                                                                                                             >  1:243420/1‑301`  
                                                                                                                                                                                                   `ATCGGTGCGGTATTCCATGAAGAGTACACACGAGATGGTCCATTTCAGTTATTTACAATCACGAAAGGAGGATAACCATATGTGTTGGGCAGCCGCAATACCTATCGCTATATCTGGCGCTATATCTGGCGCTCAGGCTCTCAGTGGTCAGAACGCTCAGGCCAAAATGATTGCCGCTCAGACCGCTGCTGGTCGTCGTCAAGCTATGGAAATCATGAGGCAGACGTACATCCAGAATGCTGACCTATCGTTGCAAGCTCGAAGTAAACTTGCGGAAGCGTCCGCCAAGTTGACCACACAa                                                                                                                      >  1:202678/1‑300`  
                                                                                                                                                                                                   `ATCGGTGCGGTATTCCATGAAGAGTACACACGAGATGGTCAATTTCAGTTATTTACAATCACGAAAGGAGGATAACCATATGTGTTGGGCAGCCGCAATACCTATCGCTATATCTGGCGCTATATCTGGCGCTCAGGCTATCAGGGGTCAGAACGCTCAGGCCAAAATGATTGCCGCTCAGACCGCTGCTGGTCGTCGTCAAGCTATGGAAATCATGAGGCAGACGAACATCCAGAATGCTGACCTATCGTTGCAAGCTCGAAGTAAACTTGAGGAAGCGTCCGCCGAGTTGACCCCACAG                                                                                                                      <  1:166391/301‑1`  
                                                                                                                                                                                                       `ataagagaaagcagGAAGAGTACACAGGAGATGGACAATTGAAGTGAGTTACAATCACGAAAGGAGGATAACCATATGTGTTGGGCAGCCGCAAGACCTATCGCTATATCTGGCGCTATATCTGGCGCTCAGGCTATCAGTGGTCAGAACGATCAGGCCAAAATGAGTGCCGCTCAGACCGCTGCTGGTCGTCGTCAAGCTATGGAAATCATGAGGCAGACGAACATCCAGAATGCTGACCTATCGTTGCAAGATCGAAGTAAACTTGAGGAAGCGTCCGCCGAGTTGACCTCACAGAACA                                                                                                                  <  1:186101/287‑1`  
                                                                                                                                                                                                       `GTGCGGTATTCCATGAAGAGTACACACGAGATGGTCAATTTCAGTTATTTACAATCACGAAAGGAGGATAACCATATGTGTTGGGCAGCCGCAATACCTATCGCTATATCTGGCGCTATATCTGGCGCTCAGGCTATCAGTGGTCAGAACGCTCAGGCCAAAATGATTGCCGCTCAGACCGCTGCTGGTCGTCGTCAAGCTATGGAAATCATGAGGCAGACGAACATCCAGAATGCTGACCTATCGTTGCAAGCTCGAAGTAAACTTGAGGAAGCGTCCGCCGAGTTGACCCCACAGAACA                                                                                                                  <  1:120649/301‑1`  
                                                                                                                                                                                                            `acagTCCATGAAGAGTACACAAGAGATGGTCAATTTCAGTTATTTACAATCACGAAAGGAGGATAACCATATGTGTTGGGCAGCCGCAATACCTATCGCTATATCTGGCGCTATATCTGGCGCTCAGGCTATCAGTGGTCAGAACGCTCAGGCCAAAATGATTGCCGCTCAGACCGCTGCTGGTCGTCGTCAAGCTATGGAAATCATGAGGCAGACGAACATCCAGAATGCTGACCTATCGTTGCAAGCTCGAAGTAAACTTGAGGAAGCGTCCGCCGAGTTGACCCCACAGAACATGCAG                                                                                                             <  1:57762/297‑1`  
                                                                                                                                                                                                            `GTATTCCATGAAGAGTACACACGAGATGGTCAATTTCAGTTATTTCCATTCACGAAAGGAGGATAACCATATGTGTTGGGCAGCCGCAATACCTATCGCTATATCTGGCACTATATCTGGCGCTCAGGCTATCAGTGGTCAGAACGCTCAGGCCAAAATGATTGCCGCTCAGACCGCTGCTGGTCGTCGTCAAGCTATGGCATTCATGAGGCAGCCGATCATCCAGAATGCTGACCTATCGTTGCCAGCTCGAAGTACACTTGAGTCAGCGTCCGCCGAGTTGACCCCACAGAACATGCAc                                                                                                             >  1:565922/1‑300`  
                                                                                                                                                                                                                `TCCATGAAGAGTACACACGAGATGGTCAATTTCAGTTATTTACAATCACGAAAGGAGGATAACCATATGTGTTGGGCAGCCGCAATACCTATCGCTATATCTGGCGCTATATCTGGCGCTCAGGCTATCAGTGGTCAGAACGCTCAGGCCAAAATGATTGCCGCTCAGACCGCTGCTGGTCGTCGTCTAGCTATGGAAATCATGAGGCAGACGAACATCCAGAATGCTGACCTATCGTTGCAAGATCGAAGTAAACTTGAGGAAGCGTCCGCCGAGTTGACCCCACAGAACATGCAGAAGG                                                                                                         >  1:248755/1‑301`  
                                                                                                                                                                                                                `TCCATGAAGAGTACACACGAGATGGTCAATTTCAGTTATTTACAATCACGAAAGGAGGATAACCATATGTGTTGGGCAGCCGCAATACCTATCGCTATATCTGGCGCTATATCTGGCGCTCAGGCTATCAGTGGTCAGAACGCTCAGGCCAAAATGATTGCCGCTCAGACCGCTGCTGGTCGTCGTCAAGCTATGGAAATCATGAGGCAGACGAACATCCAGAATGCTGACCTATCGTTGCAAGCTCGAAGTAAACTTGAGGAAGCGTCCGCCGAGTTGAACCCACAGAACATGCAGAAGG                                                                                                         >  1:180801/1‑301`  
                                                                                                                                                                                                                `TCCATGAAGAGTACACACGAGATGGTCAATTTCAGTTATTTACAATCACGAAAGGAGGATAACCATATGTGTTGGGCAGCCGCAATACCTATCGCTATATCTGGCGCTATATCTGGCGCTCAGGCTATCAGTGGTCAGAACGCTAAGGCCAAAATGATTGCCGCTCAGACCGCTGCTGGTCGTCGTCAAGCTATGGAAATCATGAGGCAGACGAACATCCAGAATGCTGACCTATCGTTGCAAGCTCGAAGTAAACTTGAGGAAGCGTCCGCCGAGTTGACCCCACAGAACATGCAGAAGG                                                                                                         >  1:494678/1‑301`  
                                                                                                                                                                                                                `TCCATGAAGAGTACACACGAGATGGTCAATTTCAGTTATTTACAATCACGAAAGGAGGATAACCATATGTGTTGGGCAGCCGCAATACCTATCGCTATATCTGGCGCTATATCTGGCGCTCAGGCTATCAGTGGTCAAAACGCTCAGGCCAAAATGATTGCCGCTCAGACCGCTGCTGGTCGTCGTCAAGCTATGGAAATCATGAGGCAGACGAACATCCAGAATGCTGACCTATCGTTGCAAGCTCGAAGTAAACTTGAGGAAGCGTCCGCCGAGTTGACCCCACAGAACATGCActgtc                                                                                                         >  1:296878/1‑296`  
                                                                                                                                                                                                                 `CCATGAAGAGTACACACGAGATGGTCAATTTCAGTTATTTACAATCACGAAAGGAGGATAACCATATGTGTTGGGCAGCCGCAATACCTATCGCTATATCTGGCGCTATATCTGGCGCTCAGGCTATCAGTGGTCAGAACGCTCAGGCCAAAATGATTGCCGCTCAGACCGCTGCTGGTCGTCGTCAAGCTATGGAAATCATGAGGCAGACGAACATCCAGAATGCTGACCTATCGTTGCAAGCTCGAAGTAAACTTGAGGAAGCGTCCGCCGAGTTGACCCCACAGAACATGCAGAAGGT                                                                                                        >  1:547698/1‑301`  
                                                                                                                                                                                                                          `GTACACACGAGATGGTCAATTTCAGTTATTTACAATCACGAAAGGAGGATAACCATATGTGTTGGGCAGCCGCAATACCTATCGCTATATCTGGCGCTATATCTGGCGCTCAGGCTATCAGTGGTCAGAACGCTCAGGCCAAAATGATTGCCGCTCAGACTGCTGCTGGTCGTCGTCAAGCTATGGAAATCATGAGGCAGACGAACATCCAGAATGCTGACCTATCGTTGCAAGCTCGAAGTAAACTTGAGGAAGCGTCAGACGAGATGACCCAAAAGAACATGCAGAAGGTCCAAGCAAT                                                                                               >  1:662611/1‑301`  
                                                                                                                                                                                                                          `GTACACACGAGATGGTCAATTTCAGTTATTTACAATCACGAAAGGAGGATAACCATATGTGTTGGGCAGCCGCAATACCTATCGCTATATCTGGCGCTATATCTGGCGCTCAGGCTATCAGTGGTCAGAACGCTCAGGCCAAAATGATTGCCGCTCAGACCGCTGCTGGTCGTCGTCAAGCTATGGAAATCATGAGGCAGACGAACATCCAGAATGCTGACCTATCGTTTCAAGCTCGAAGTAAACTTGAGGAAGCGTCCGCCGAGTTGACCCCACAGAACATGCAGAAGGTCCAAGCTAT                                                                                               >  1:388318/1‑301`  
                                                                                                                                                                                                                          `GTACACACGAGATGGTCAATTTCAGTTATTTACAATCACGAAAGGAGGATAACCATATGTGTTGGGCAGCCGCAATACCTATCGCTATATCTGGCGCTATATCTGGCGCTCAGGCTATCAGTGGTCAGAACGCTCAGGCCAAAATGATTGCCGCTCAGACCGCTGCTGGTCGTCGTCAAGCTATGGAAATCATGAGGCAGACGAACATCCAGAATGCTGACCTATCGTTGCAAGCTCGAAGTAAACTTGAGGAAGCGTCCGCCGCGTTGACCCCACAGAACATGCAGAAGGTCCAAGCTAT                                                                                               >  1:536206/1‑301`  
                                                                                                                                                                                                                          `GTACACACGAGATGGTCAATTTCAGTTATTTACAATCACGAAAGGAGGATAACCATATGTGTTGGGCAGCCGCAATACCTATCGCTATATCTGGCGCTATATCTGGCGCTCAGGCTATCAGTGGTCAGAACGCTCAGGCCAAAATGATTGCCGCTCAGACAGCTGCTGGTCGTCGTCAAGCTATGGAAATCATGAGGCAGACGAACATCCAGAATGCTGACCTATCGTTGCAAGCTCGAAGTAAACTTGAGGAAGCGTCCGCCGAGTTGCCCCCACAGAACATGCAGAAGGTCCAAGCTAT                                                                                               >  1:393301/1‑301`  
                                                                                                                                                                                                                               `aACGAGATAGTCAATTTCAGTTATTTACAATCACGAAAGGAGGATAACCATATGTGTTGGGCAGCCGCAATACCTAACGCTATATCTGGCGCTATATCTGGCGCTCAGGCTATAAGTGGGCAGAACGCTCAGGCCAAAATGATGGCCGCTAAAACCTCGGCTGGTAGTCGTCAAGCTATGGAAATCATGAGGCAGACGAACATCCAGAATGCTGACCTATCGTTGCAAGCTCGAAGTAAACTTGAGGAAGCGTCCGCCGAGTTGACCCCAAAGAACATGCAGAAGGTCCAAGCTATTGGGT                                                                                          <  1:647003/300‑1`  
                                                                                                                                                                                                                               `CACGAGATGGTCAATTTCAGTTATTTACAATCACGAAAGGAGGATAACCATATGTGTTGGGCAGCCGCAATACCTATCGCTATATCTGGCGCTATATCTGGCGCTCAGGCTATCAGTGGTCAGAACGCTCAGGCCAAAATGATTGCCGCTCAGACCGCTGCTGGTCGTCGTCAAGCTATGGAAATCATGAGGCAGACGAACATCCAGAATGCTGACCTATCGTTGCAAGCTCGAAGTAAACTTGAGGAAGCGTCCGCCGAGTTGACCCCACAGAACATGCAGAAGGTCCAAGCTATTGGGT                                                                                          <  1:37481/301‑1`  
                                                                                                                                                                                                                               `CACGAGATGGTCAATTTCAGTTATTTACAATCACGAAAGGAGGATAACCATATGTGTTGCGCAGCCGCAATACCTATCGCTATATCTGGCGCTATATCTGGCGCTCAGGCTATCAGTGGTCAGAACGCTCAGGCCAAAATGATTGCCGCTCAGACCGCTGCTGGTCGTCGTCAAGCTATGGAAATCATGAGGCAGACGAACATCCAGAATGCTGACCTATCGTTGCAAGCTCGAAGTAAACTTGAGGAAGCGTCCGCCGAGTTGACCCCACAGAACATGCAGAAGGTCCAAGCTATTGGGT                                                                                          <  1:232270/301‑1`  
                                                                                                                                                                                                                               `CACGAGATGGTCAAGTTCAGTTATTTACAATCACGAAAGGAGGATAACCATATGTGTTGGGCAGCCGCAATACCTATCGCTATATCTGGCGCTATATCTGGCGCTCAGGCTATCAGTGGTCAGAACGCTCAGGCCAAAATGATTGCCGCTCAGACCGCTGCTGGTCGTCGTCAAGCTATGGAAATCATGAGGCAGACGAACATCCAGAATGCTGACCTATCGTTGCAAGCTCGAAGTAAACTTGAGGAAGCGTCCGCCGAGTTGACCTCACAGAACATGCAGAAGGTCCAAGCTATTGGGT                                                                                          <  1:451502/301‑1`  
                                                                                                                                                                                                                                    `GATTGTCAATTGCAGTGATTTACAATCACGAAAGGAGGATAACCATATGTGTGGGGCAGCCGCAATACCTATCGCTATATCTGGCGCTATATCTGGCGCTCAGGCTATCAGTGGTCAGAACGCTCAGGCCAAAATGAGTGCCGCTCAGACCGCTTCTGGTCGTCGTCAAGCTATGGAAATCATGATGCAGACGAACATCCAGAATGCTGACCTATCGTTGCAAGCTCGAAGTAAACGTGAGGAAGCGTCCGCCGAGTTGACCCCACAGAACATGCAGAAGGTCCAAGCTATTGGGTCTATC                                                                                     <  1:237290/301‑1`  
                                                                                                                                                                                                                                                  `GTTATTTACAATCACGAAAGGAGGATAACCATATGTGTTGGGCAGCCGCAATACCTATCGCTATATCTGGCGCTATATCTGGCGCTCAGGCTATCAGTGGTCAGAACGCTCAGGCCAAAATGATTGCCGCTCATACCGCTGCTGGTCGTCGTCAAGCTATGGAAATCATGAGGCAGACGAACATCCAGAATGCTGACCTATCGTTGCAAGCTCGAAGTAAACTTGAGGAAGCGTCCGCCGAGTTGACCCCACAGAACATGCAGAAGGTCCAAGCTATTGGGTCTATCCGAGCGGCTATCGG                                                                       >  1:398561/1‑301`  
                                                                                                                                                                                                                                                    `TATTTACAATCACGAAAGGAGGATAACCATATGTGTTGGGCAGCCGCACTACCTATCGCTATATCTGGCGCTATATCTGGCGCTCAGGCTATCAGTGGTCAGAACGCGCAGGCCAAAATGATTGCCGCTCAGACCGCTGCTGGTCGTCGTCAAGCTATGGAAATCATGAGGCAGACGAACATCCAGAATGCTGACCTATCGTTGCAAGCTCGAAGTAAACTTGAGGAAGCGTCCGCCGAGTTGACCTCACAGAACATGCAGAAGGTCCAAGCTATTGGGTCTATCCGAGCGGCTATCGGAG                                                                     <  1:668340/301‑1`  
                                                                                                                                                                                                                                                    `TATTTACAAACACGAAAGGAGGATAACCATATGTGTTGGGCAGCCGCAATACCTATCGCTATATCTGGCGCTATAGCTGGCGCTCAGGCGATCAGTGGTCAGAACGCTCAGGCCAAAATGATTGCCGCTCAGACCGCTGCTGGTCGTCGTCAAGCTATGGAAATCATGAGGCAGACGAACATCCAGAATGCTGACCTATCGTTGCAAGCTCGAAGTAAACTTGAGGAAGCGTCCGCCGAGTTGACCTCACAGAACATGCAGAAGGTCCAAGCTATTGGGTCTATCCGAGCGGCTATCGGAG                                                                     <  1:416570/301‑1`  
                                                                                                                                                                                                                                                          `aAATCACGAAAGGAGGAGAACCATATGTGTTGGGCAGCCGCAATACCTATCGCTATATCTGGAGCTATATCTGGCGCTCAGGCTATCAGTGGTCAGAACGCTCAGGCCAAAATGATTGCCGCTCAGACCGCTGCTGGTCGTCGTCAAGCTATGGAAATCATGAGGCAGACGAACATCCAGAATGCTGACCTATCGTTGCAAGCTCGAAGTAAACTTGAGGAAGCGTCCGCCGAGTTGACCCCACAGAACATGCAGAAGGTCCAAGCTATTGGGTCTATCCGAGCGGCTATCGGAGAGAGTA                                                               <  1:369240/300‑1`  
                                                                                                                                                                                                                                                              `CACGAAAGGAGGATAACCATATGTGTTGGGCAGCCGCAATACCTATCGCTATTTCTGGCGCTATATCTGGCGCTCAGGCTATCAGTGGTCAGAACGCTCAGGCCAAAATGATTGCCGCTCAGACCGCTGCTGGTCGTCGTCAAGCTATGGAAATCATGAGGCAGACGAACATCCAGAATGCTGACCTATCGTTGCAAGCTCGAAGTAAACTTGAGGAAGCGTCCGCCGAGTTGACCCCACAGAACATGCAGAAGGTCCAAGCTATTGGGTCTATCCGAGCGGCTATCGGAGAGAGTATGCT                                                           <  1:290599/301‑1`  
                                                                                                                                                                                                                                                              `CACGAAAGGAGGATAACCATATGTGTTGGGCAGCCGCAATACCTATCGCTATATCTGGCGCTATATCTGGCGCTCAGGCTATCAGTGGTCAGAACGCTCAGGCCAAAATGATTGCCGCTCAGACCGCTGCTGGTCGTCGTCAAGCTATGGAAATCATGAGGCAGACGAACATCCAGAATGATGACCTATCGTTGCAAGCTCGAAGTAAACTTGAGGAAACGTCCTCCGAGTTGACCCCACAGAACATGCAGAAGGTCCAAGCTATTGGGTCTATCCGAGCGGCTATCGGAGAGAGTActgt                                                           >  1:362215/1‑297`  
                                                                                                                                                                                                                                                              `CACGAAAGGAGGATAACCATATGTGTTGGGCAGCCGCAATACCTATCGCTATATCTGGCGCTATATCTGGCGCTCAGGCTATCAGTGGTCAGAACGCTCAGGACAAAATGATTGCCGCTCAGACCGCTGCTGGTCGTCGTCAAGCTATGGAAATCATGAGGCAGACGAACATCCAGAATGCTGACCTATCGTTGCAAGCTCGAAGTAAACTTGAGGAAGCGTCCGCCGAGTTGACCCCACAGAACATGCAGAAGGTCCAAGCTATTGGGTCTATCCGAGCGGCTATCGGAGAGAGTATGCT                                                           >  1:571023/1‑301`  
                                                                                                                                                                                                                                                              `CACGAAAGGAGGATAACCATATGTGTTGGGCAGCCGCAATACCTATCGCTATATCTGGCGCTATATCTGGCGCTCAGACTATCAGTGGTCAGAACGCTCAGGCCAAAATGATTGCCGCTCAGACCGCTTCTGGTCGTCGTCAAGCTATGGAAATCATGAGGCAGACGAACATCCAGAATGCTGACCTATCGTTGCAAGCTCGAAGTAAACTTGAGGAAGCGTCCGCCGAGTTGACCCCACAGAACATGCAGAAGGTCCAAGCTATTGGGTCTATCCGAGCGGCTATCGGAGAGAGTATGCT                                                           <  1:208399/301‑1`  
                                                                                                                                                                                                                                                              `CACGAAAGGAGGATAACCATATGTGTTGGGCAGCCGCAATACCTATCGCTATATCTGGCGCGATATCTGGCGCTCAGGCTATCAGTGGTCAGAACGCTCAGGCCAAAATGATTGCCGCTCAGACCGCTGCTGGTCGTCGTCAAGCTATGGAAATCATGAGGCAGACGAACATCCAGAATGCTGACCTATCGTTGCAAGCTCGAAGTAAACTTGAGGAAGCGTCCGCCGAGTTGACCCCACAGAACATGCAGAAGGTCCAAGCTATTGGGTCTATCCGAGCGGCTATCGGAGAGAGTATGCT                                                           <  1:135347/301‑1`  
                                                                                                                                                                                                                                                                `CGAAAGGAGTGTAACCATATGTGTTGGGCAGCCGCAATACCTATCGCTATATCTGGCGCTATATCAGGCGCTCAGGCTATCAGTGGTCAGAACGCTCAGGCCAAGATGATTGCCGCTCAGACCGCTGCTGGTCGTCGTCAAGCTATGGAAATCATGAGGCAGACGAACATCCAGAATGCTGACCTATCGTTGCAAGCTCGAAGTAAACTTGAGGAAGCGTCCGCCGAGTTGACCCCACAGAACATGCAGAAGGTCCAAGCTATTGGGTCTATCCGAGCGGCTATCGGAGAGAGTATGCTTG                                                         <  1:459508/301‑1`  
                                                                                                                                                                                                                                                                `CGAAAGGAGGATAACCATATGTGTTGGGCAGCCGCAATACCTATCGCTATATCTGGCGCTATATCTGGCGCTCAGGCTATCAGTGGTCAGAACGCTCAGGCCAAAATGATTGCCGCTCAGACCGCTGCTGGTCGTCGTCAAGCTATGGAAATCATGAGGCAGACGAACATCCAGAATGCTGACCTATCGTTGCAAGCTCGAAGTAAACTTGAGGAAGCGTCCGCCGAGTTGACCCCACAGAACATGCAGAAGGTCCAAGCTATTGGGTCTATCCGAGCGGCTATCGGAGAGAGTATGCTTG                                                         <  1:431988/301‑1`  
                                                                                                                                                                                                                                                                    `AGGAGGATAACCATATGTGTTGGGAAGCCGCAATACCGATCGCTATATCTGGCGCTATATCTGGCGCTCAGGCTATCAGTGGTCAGAACGCTCAGGCCAAAATGATTGCCGCTCAGACCGCTGCTGGTCGTCGTCAAGCTATGGAAATCATGAGGCAGACGAACATCCAGAATGCTGACCTATCGTTGCAAGCTCGAAGTAAACTTGAGGAAGCGTCCGCCGAGTTGACCTCACAGAACATGCAGAAGGTCCAAGCTATTGGGTCTATCCGAGCGGCTATCGGAGAGAGTATGCTTGAAGG                                                     <  1:421301/301‑1`  
                                                                                                                                                                                                                                                                     `GGAGGATAACCATATGTGTTGGGAAGCCGCAATACCTATCGCTATATCTGGCGCTATATCTGGCGCTCAGGCTATCAGTGGTCAGAACGATCAGGCCAAAATGATTGCCGCTCAGACCGCTGCTGGTCGTCGTCAAGCTATGGAAATCATGAGGCAGACGAACATCCAGAAGGCTAACCTATCGTTGCAAGCTCGAAGTAAACTTGAGGAAGCGTCCGCCGAGTTGACCCAACAGAACATGCAGAAGGTCCAAGCTATTGGGTCTATCCGAGCGGCTATCGGAGAGAATATGCTTGAAGGT                                                    <  1:355207/301‑1`  
                                                                                                                                                                                                                                                                        `GGATAACCATATGTGTTGGGCAGCCTCAATACCTATCGCTATATCTGGCGCTATATCTGGCGCTCAGGCTATCAGTGGTCAGAACGCTCAGGCCAAAATGATTGCCGCTCAGACCGCTGCTGGTCGTCGTCAAGCTATGGAAATCATGAGGCAGACGAACATCCAGAATGCTGACCTATCGTTGCAAGCTCGAAGTAAACTTGAGGAAGCGTCCGCCGAGTTGACCCCACAGAACATGCAGAAGGTCCAAGCTATTGGGTCTATCCGAGCGGCTATCGGAGCGAGTATGCTTGAAGGTTCC                                                 >  1:488907/1‑301`  
                                                                                                                                                                                                                                                                         `GATAACCATATGTGTTGGGCAGCCGCAATACCTATCGCTATATCTTGCGCTATATCTGGCGCTCAGGCTACCAGTGGTCAGAACGCTCAGGCCAAAATGATTGCCGCTCAGACCGCTGCTGGTCGTCGTCAAGCTATGGAAATAAGGAGGCAGACGAACATCCAGAATGCTGACCTATCGTTGCAAGCTCGAAGTAAACTTGAGGAAGCGTCCGCCGAGTTGACCCCACAGAACATGCAGAGGGTCCAAGCTATTGGGTCTATCCGAGCGGCTATCGGGGAGAGTCTGCATGAAGGTTCCc                                                >  1:629141/1‑300`  
                                                                                                                                                                                                                                                                               `CATATGTGTTGGGCAGCCGCAATACCTATCGCTATATCTGGCGCTATATCTGGCGCTCAGGCTATCAGTGGTCAGAACGCTCAGGCCAAAATGATTGCCGCTCAGACCGCTGCTGGTCGTCGTCAAGCTATGGAAATCATGAGGCAGACGAACATCCAGAATGCTGACCTATCGTTGCAAGCTCGAAGTAAACTTGAGGAAGCGTCCGCAGAGTTGACCTCACAGAACATGCAGAAGGTCCAAGCTATTGGGGCTATCCGAGCGGCTATCGGAGAGAGTATGCTTGAAGGTTTCTCAATGG                                          >  1:329640/1‑301`  
                                                                                                                                                                                                                                                                                  `ATGTGTTGGGCAGCCGCAATACCTATCGCTATATCTGGCGCTATATCTGGCGCTCAGGCTATCAGTGGTCAGAACGCTCAGGCCAAAATGATTGCCGCTCAGACCGCTGCTGGTCGTCGTCAAGCTATGGAAATCATGAGGCAGACGAACATCCAGAATGCTGACCTATCGTTGCAAGCTCGAAGTAAACTTGAGGAAGCGTCCGCCGAGTTGACCCCACAGAACATGCAGAAGGTCCAAGCTATTGGGTCTATCCGAGCGGCTATCGGAGAGAGTATGCTTGAAGGTTCCTCAATGGACC                                       <  1:505474/301‑1`  
                                                                                                                                                                                                                                                                                    `GTGTTGGGCAGCCGCAATACCTATCGCTATATCTTGCGCTATATCTGGCGCTCAGGCTATCAGTGGACAGAACGCTCAGGCCAAAATGATTGCCGCTCAGACCGCTGCTGGTCGTCGTCCAGCTATGGAAATCATGAGGCAGACGAACATCCAGAATGCTGCCCTATCGTTGCAAGCTCGCAGTACACTTGAGGAAGCGTCCGCCGAGTTGACCCCACAGAACATGCAGAAGGTCCAAGCTATTGGGTCTATCCGAGCGGCTATCGGATAGAGTATGCTTGAAGGTTCCTCAATGTACCGC                                     >  1:524003/1‑301`  
                                                                                                                                                                                                                                                                                    `GTGTTGGGCAGCCGCAATACCTATCGCTATATCTGGCGCTATATCTGGCGCTCAGGCTATCAGTGGTCAGAACGCTCAGGCCAAAATGATTGCCGCTCAGACCGCTGCTGGTCGTCGTCAAGCTATGGAAATCATGAAGCAGACGAACATCCAGAATGCTGACCTATCGTTGCAAGCTCGAAGTAAACTTGAGGAAGCGTCCGCCGAGTTGACCCCACAGAACATGCAGAAGGTCCAAGCTATTGGGTCTATCCGAGCGGCTATCGGAGCGAGTATGCTTGAAGGTTCCTCAATGGctgtt                                     >  1:285243/1‑296`  
                                                                                                                                                                                                                                                                                      `gagatgtgtataagggacagATCGCTATATCTGGCGCTATATCTGGCGCTCAGGCTATCAGTGGTCAGAACGCGCAGGCCAAAATGATTGCCGCTCAGACCGATGCTGGTCGTCGTAAAGCTATGGAAATCATGAGGCAGACGAACATCCAGAATGCTGAACGATCGTTGCAAGCTCGAAGTAAACTTGAGGAAGCGTCCGCCGAGTTGACACCACAGAACATGCAGAAGGACCAAGCTATTGGGTCTAGCCGAGCGGCTATCGGAGAGAGTATGCTTGAAGGTTCCTCAATGGACCGCAT                                   <  1:636057/281‑1`  
                                                                                                                                                                                                                                                                                          `GGCAGCCGCAATACCTATCGCTATATCTGGCGCTATATCTGGCGCTCAGGCTATCAGTGGTCAGAACGCTCAGGCCAAAATGATTGCCGCTCAGACCGCTGCTGGTCGTCGTCAAGCTATGGAAATCATGAGGCAGACGAACATCCAGAATGCTGACCTATCGTTGCAAGCTCGAAGTAAACTTGAGGAAGCGTCCGCCGAGTTGACCCCACAGAACATGCAGAAGGTCCAAGCTATTGGGTCTATCCGAGCGGCTATCGGAGAGAGTATGCTTGAAGGTTCCTCAATGGACCGCATTAAG                               <  1:174201/301‑1`  
                                                                                                                                                                                                                                                                                           `aaagagacagTACCTATCGCTATATCTGGCGCTATATCTGGCGCTCAGGCTATCAGTGGTCAGAACGCTCAGGCCAAAATGATTGCCGCTCAGACCGCTGCTGGTCGTCGTCAAGCTATGGAAATCATGAGGCAGACGAACATCCAGAATGCTGACCTATCGTTGCAAGCTCGAAGTAAACTTGAGGAAGCGTCCGCCGAGTTGACCCCACAGAACATGCAGAAGGTCAAAGCTATTGGGTCTATCCGAGCGGCTATCGGAGAGAGTATGCTTGAAGGTTCCTCAATGGACCGCATTAAGC                              <  1:38017/291‑1`  
                                                                                                                                                                                                                                                                                           `GCAGCCGCAATACCTATCGCTATATCTGGCGCTATATCTGGCGCTCAGGCTATCAGTGGTCAGAACGCTCAGGCCAAAATGATTGCCGCTCAGACCGCTGCTGGTCGTCGTCAAGCTATGGAAATCATGAGGCAGACGAACATCCAGAATGCTGACCTATCGTTGCAAGCTCGAAGTAAACTTGAGGAAGCGTCCGCCGAGTTGACCCCACAGAACATGCAGAAGGTCCAAGCTATTGGGTCTATCCGAGCGGCTATCGGAGAGAGTATGCTTGAAGGTTCCTCAATGGACCGCATTAAGC                              <  1:517904/301‑1`  
                                                                                                                                                                                                                                                                                                `CGCAATACCTATCGCTATATCTGGCGCTATATCTGGCGCTCAGGCTATCAGTGGTCAGAACGCTCAGGCCAAAATGCTTGCCGCTCAGACCGCTGCTGGTCGTCGTCAAGCTATAGAAATCATGAGGCAGACGAACATCCAGAATGCTGACCTATCGTTGCAAGCTCGAAGTAAACTTGAGGAAGCGTCCGCCGAGTTGACCCCACAGAACATGCAGAAGGTCCAAGCTATTGGGTCTATCCGAGCGGCTATCGGAGAGAGTATGATTGAAGGTTCCTCAATGGACCGCATTAAGCGAGTa                         >  1:307855/1‑300`  
                                                                                                                                                                                                                                                                                                     `aACCTATCGCTATAGCTGGCGCTATATCTGGCGCTCAGGCTATCAGTTGTCAGAACGATAAGGCCAAAATGATTGAAGCTCAGACCGGTGCTGGTCGTCGTAAAGCTATGGAAATCATGAGGCAGACGAACATCCAGAATGCTGACCTATCGTTGCAAGCTCGAAGTAAACTTGAGGAAGCGTCCGCCGAGTTGAACCCACAGAACATGCAGAAGGTCCAAGCTATTGGGTCTATCCGAGCGGCTATCGGAGAGAGTATGCTTGAAGGTTCCTCAATGGACCGCATTAAGCGAGTCACAGA                    <  1:100163/300‑1`  
                                                                                                                                                                                                                                                                                                     `TACCTATCGCTATATCTGGCGCTATATCTGGCGCTCAGGCTATCAGTGGTCAGAACGCTCAGGCCAAAATGATTGCCGCTCAGACCGCTGCTGGTCGTCGTCAAGCTATGGAAATCATGAGGCAGACGAACATCCAGAATGCTGACCTATCGTTGCAAGCTCGAAGTAAACTTGAGGAAGCGTCCGCCGAGTTGACCCCACAGAACATGCAGAAGGTCCAAGCTATTGGGTCTATCCGAGCGGCTATCGGAGAGAGTATGCTTGAAGGTTCCTCAATGGACCGCATTAAGCGAGTCACAGA                    <  1:213908/301‑1`  
                                                                                                                                                                                                                                                                                                      `ACCTATCGCTCTATCTGGCGCTATATCTGGCGCTCAGGCTAACAGTGGACAGAACGCTCAGGCCAAAATGATTGCCGCTCAGACCGATGCTGGTCGTCGTCATGCTATGGAAATCATGAGGCAGACGAACATCCAGAATGCTGATCTATCGTTGCAAGCTCGAAGTAAACTTGAGGAAGCGTCCGACGAGTTGCCCCCACAGAACATGCAGAAGGTCCAAGCTCTTGGGTCTATCCGAGCGGCTATCGGAGAGCGTATGCTTGAAGGTTCCCACATGGAACGCATTTAGCGACTCACAaca                   >  1:313725/1‑298`  
                                                                                                                                                                                                                                                                                                          `cgCGCTATATCTGTCGCTATATCTGGCGCTCAGTCTATCAGTGGTCAGAACGCTCAGGCCAAAATGATTGCCGCTCAGACCGCTGCTGGTCGTCGTCAAGCTATGGAAATCATGAGGCAGACGAACATCCAGAATGCTGACCTATCGTTGCAAGCTCGAAGTAAACTTGAGGAAGCGTCCGCCGAGTTGACCCCACAGAACATGCAGAAGGTCCAAGCTATTGGGTCTATCCGAGCGGCTATCTGAGAGAGTATGCTTTAAGGTTCCTCAATGGACCGCATTAAGCGAGTCACAGAAGGAC               <  1:500555/299‑1`  
                                                                                                                                                                                                                                                                                                          `ATCGCTATATCTGGCGCTATATCTGGCGCTCAGGCTATCAGTGGTCAGAACGCTCAGGCCAAAATGATTGCCGCTCAGACCGCTGCTGGTCGTCGTCAAGCTATGGAAATCATGAGGCAGACGAACATCCAGAATGCTGACCTATCGTTGCAAGCTCGAAGGAAACTTGAGGAAGCGTCCGCCGAGTTGACCCCACAGAACATGCAGAAGGTCCAAGCTATTGGGTCTATCCGAGCGGCTATCGGAGAGAGTATGCTTGAAGGTTCCTCAATGGACCGCATTAAGCGAGTCACAGAAGGAC               <  1:410771/301‑1`  
                                                                                                                                                                                                                                                                                                           `TCGCTATATCTGGCGCTATATCTGGCGCTCAGGCTATCAGTGGTCAGAACGCTCAGGCCAAAATGATTGCCGCTCAGACCGCTGCTGGTCGTCGTCAAGCTATGGAAATCATGAGGCAGACGAACATCCAGAATGCTGACCTATCGTTGCAAGCTCGAAGTAAACTTGAGGAAGCGTCCGCCGAGTTGACCCCACAGAACATGCAGAAGGTCCAAGCTATTGGGTCTATCCGAGCGGCTATCGGAGAGAGTATGCTTGAAGGTTCCTCCATGGACCGCATTAAGCGAGTCACAGAAGGCCA              >  1:86592/1‑301`  
                                                                                                                                                                                                                                                                                                            `CGCTATATCTGGCGCTATATCTGGCGCTCAGGCTATCAGTGGTCAGAACGCTCAGGCCAAAATGATTGCCGCTCAGACCGCTGCTGGTCGTCGTCAAGCTATGGAAATCATGAGGCAGACGAACATCCAGAATGCTGACCTATCGTTGCAAGCTCGAAGTAAACTTGAGGAAACGTCCGCCGAGTTGACCCCACAGAACATGCAGAAGGTCCAAGCTATTGGGTCTATCCGAGCGGCTATCGGAGAGAGTATGCTTGAAGGTTCCTCAATGGACCGCATTAAGCGctgtctcttatacaca             >  1:322706/1‑285`  
                                                                                                                                                                                                                                                                                                                `ATATCTGGCGCTATATCTGGCGCTCAGGCTATCAGTGGTCAGAACGCTCAGGCCAAAATGATTGCCGCTCAGACCGCTGCTGGTCGTCGTCAAGCTATGGAAATCATGAGGCAGACGAACATCCAGAATGCTGACCTATAGTTGCAAGCTCGAAGTAAACTTGAGGAAGCGTCCGCCGAGTTGACCCCACAGAACATGCAGAAGGTCCAAGCTATTGGGTCTATCCGAGCGGCTATCGGAGAGAGTATGCTTGAAGGTTCCTCAATGGACCGCATTAAGCGAGTCACAGAAGGACAGTTCA         >  1:226896/1‑301`  
                                                                                                                                                                                                                                                                                                                  `ATCTGGCGCTATATCTGGCGCTCAGGCTATCAGTGGTCAGAACGCTCAGGCCAAAATGATTGCCGCTCAGACCGCTGCTGGTCGTCGTCAAGCTATGGAAATCATGAGGCAGACGAACATCCATAATTCTGACCTATCGTTGCAAGCTCGAAGTAAACTTGAGGAAGCGTCCGCCGAGTTGACCCCACAGAACATGCAGAAGGTCCAAGCTATTGGGTCTATCCGAGCGGCTATCGGAGAGAGTATGCTTGAAGGTTCCTCAATGGACCGCATTAAGCGAGTCACAGAAGGACAGTTCATT       >  1:93347/1‑301`  
                                                                                                                                                                                                                                                                                                                      `GGCGCTATATCTGGCGCTCAGGCTATCAGTGGTCAGAACGCTCAGGCCAAAATGATTGCCGCTCAGACCGCTGCTGGTCGTCGTCAAGCTATGGAAATCATGAGGCAGACGAACATCCAGAATGCTGACCTATCGTTGCAAGCTCGAAGTAAACTTGAGGAAGCGTCCGCCGAGTTGACCCCACAGAACATGCAGAAGGTCCAAGCTATTGGGTCTATCCGAGCGGCTATCGGAGAGAGTATGCTTGAAGGTTCCTCAATGGACCGCATTAAGCGctgtctcttatacacatctccgagcc   >  1:512577/1‑275`  

                                                                                                                                                                                                                                                                                                            `||||||||||||`                                                                                                                                                                                                                                                                                                              
`CTAGCTATCGGTGGTAACTGCGGGGACCAGTGCTGGTTCGTTACGAGCGACCAAGTGTGGCGACTTAGTGGAAAGGCTAAGCGAAAGTTCCGTAAGTTAATCATGGAGTATCGCGATAAGATGCTTGAGAAGTATGATACTCTTTGGAATTACGTATGGGTAGGCAATACGTCCCACATTCGTTTCCTCAAGACTATCGGTGCGGTATTCCATGAAGAGTACACACGAGATGGTCAATTTCAGTTATTTACAATCACGAAAGGAGGATAACCATATGTGTTGGGCAGCCGCAATACCTAT‑‑‑‑‑‑‑‑‑‑‑‑‑‑‑‑‑‑‑‑‑‑‑‑‑‑‑‑‑‑‑‑‑‑‑‑‑‑‑‑‑‑‑‑‑‑‑‑‑‑‑‑‑‑‑‑‑‑‑‑‑‑‑‑‑‑‑‑‑‑‑‑‑‑‑‑‑‑‑‑‑‑‑‑‑‑‑‑‑‑‑‑‑‑‑‑‑‑‑‑‑‑‑‑‑‑‑‑‑‑‑‑‑‑‑‑‑‑‑‑‑‑‑‑‑‑‑‑‑‑‑‑‑‑‑‑‑‑‑‑‑‑‑‑‑‑‑‑‑‑‑‑‑‑‑‑‑‑‑‑‑‑‑‑‑‑‑‑‑‑‑‑‑‑‑‑‑‑‑‑‑‑‑‑‑‑‑‑‑‑‑‑‑‑‑‑‑‑‑‑‑‑‑‑‑‑‑‑‑‑‑‑‑‑‑‑‑‑‑‑‑‑‑‑‑‑‑‑‑‑‑‑‑‑‑‑‑‑‑‑‑‑‑‑‑‑‑‑‑‑‑‑‑‑‑‑‑‑‑‑‑‑‑‑‑‑‑‑‑‑‑‑‑‑‑‑‑‑‑‑‑‑‑‑‑‑‑‑‑‑‑‑‑‑‑‑‑‑‑‑‑‑‑‑‑‑‑‑‑‑‑‑  >  NC_001604.1/27454‑27753`  
`‑‑‑‑‑‑‑‑‑‑‑‑‑‑‑‑‑‑‑‑‑‑‑‑‑‑‑‑‑‑‑‑‑‑‑‑‑‑‑‑‑‑‑‑‑‑‑‑‑‑‑‑‑‑‑‑‑‑‑‑‑‑‑‑‑‑‑‑‑‑‑‑‑‑‑‑‑‑‑‑‑‑‑‑‑‑‑‑‑‑‑‑‑‑‑‑‑‑‑‑‑‑‑‑‑‑‑‑‑‑‑‑‑‑‑‑‑‑‑‑‑‑‑‑‑‑‑‑‑‑‑‑‑‑‑‑‑‑‑‑‑‑‑‑‑‑‑‑‑‑‑‑‑‑‑‑‑‑‑‑‑‑‑‑‑‑‑‑‑‑‑‑‑‑‑‑‑‑‑‑‑‑‑‑‑‑‑‑‑‑‑‑‑‑‑‑‑‑‑‑‑‑‑‑‑‑‑‑‑‑‑‑‑‑‑‑‑‑‑‑‑‑‑‑‑‑‑‑‑‑‑‑‑‑‑‑‑‑‑‑‑‑‑‑‑‑‑‑‑‑‑‑‑‑‑‑‑‑‑‑‑‑‑‑‑‑‑‑‑‑‑‑‑‑‑‑‑‑‑‑‑‑‑‑‑‑‑‑‑‑‑‑‑‑‑‑‑‑‑‑‑‑‑‑‑‑‑‑‑‑‑‑CGCTATATCTGGCGCTCAGGCTATCAGTGGTCAGAACGCTCAGGCCAAAATGATTGCCGCTCAGACCGCTGCTGGTCGTCGTCAAGCTATGGAAATCATGAGGCAGACGAACATCCAGAATGCTGACCTATCGTTGCAAGCTCGAAGTAAACTTGAGGAAGCGTCCGCCGAGTTGACCTCACAGAACATGCAGAAGGTCCAAGCTATTGGGTCTATCCGAGCGGCTATCGGAGAGAGTATGCTTGAAGGTTCCTCAATGGACCGCATTAAGCGAGTCACAGAAGGACAGTTCATTCGGGA  >  NC_001604.1/27754‑28053`

  

Alignment Legend

Aligned base mismatch/match (shaded by quality score): `ATCG/ATCG` `< 3 ≤` `ATCG/ATCG` `< 10 ≤` `ATCG/ATCG` `< 20 ≤` `ATCG/ATCG` `< 34 ≤` `ATCG/ATCG` `< 38 ≤` `ATCG/ATCG`

Unaligned base: `atcg`    Masked matching base: `atcg`    Alignment gap: `‑`    Deleted base: `‑`

Reads not counted as support for junction

read\_name Not counted due to insufficient overlap past the breakpoint.

read\_name Not counted due to not crossing MOB target site duplication.
